# What are the barriers to, and facilitators of, implementing and receiving MHPSS programmes delivered to populations affected by humanitarian emergencies? A qualitative evidence synthesis

**DOI:** 10.1017/gmh.2018.12

**Published:** 2018-06-01

**Authors:** Kelly Dickson, Mukdarut Bangpan

**Affiliations:** Department of Social Science, University College London Institute of Education, London WC1H 0AL, UK

**Keywords:** Humanitarian emergencies, Interventions, Low- and middle-income countries, Mental health and psychosocial programmes, Programme implementation, Qualitative evidence synthesis

## Abstract

**Background.:**

Humanitarian emergencies can impact people's psychosocial well-being and mental health. Providing mental health and psychosocial support (MHPSS) is an essential component of humanitarian aid responses. However, factors influencing the delivery MHPSS programmes have yet to be synthesised. We undertook a systematic review on the barriers to, and facilitators of, implementing and receiving MHPSS programmes delivered to populations affected by humanitarian emergencies in low- and middle-income countries.

**Methods.:**

A comprehensive search of 12 bibliographic databases, 25 websites and citation checking was undertaken. Studies published in English from 1980 onwards were included if they contained evidence on the perspectives of adults or children who had engaged in or programmes providers involved in delivering, MHPSS programmes in humanitarian settings. Thirteen studies were critically appraised and analysed thematically.

**Results.:**

Community engagement was a key mechanism to support the successful implementation and uptake of MHPSS programmes. Establishing good relationships with parents may also be important when there is a need to communicate the value of children and young people's participation in programmes. Sufficient numbers of trained providers were essential in ensuring a range of MHPSS programmes were delivered as planned but could be challenging in resource-limited settings. Programmes need to be socially and culturally meaningful to ensure they remain appealing. Recipients also valued engagement with peers in group-based programmes and trusting and supportive relationships with providers.

**Conclusion.:**

The synthesis identified important factors that could improve MHPSS programme reach and appeal. Taking these factors into consideration could support future MHPSS programmes achieve their intended aims.

## Background

More than 128.6 million people across 33 countries require humanitarian assistance (OCHA, 2017). Populations affected by natural and man-made humanitarian emergencies can present with a range of mental health and psychosocial support (MHPSS) needs (Steel *et al.* 2009; Roberts & Browne, 2011; Tang *et al.* 2014). Although many retain good psychological health by drawing on social and individual protective factors, pre-existing mental health and psychosocial status, concurrent life events, and a lack of buffering from stressors caused by humanitarian emergencies can result in significant mental health and psychosocial difficulties (Lock *et al.* 2012; Silove, 2013). A key recommendation of international humanitarian aid guidelines is to ensure mental health and psychosocial programmes are made available to local communities in disaster and post-disaster settings (IASC, 2007; WHO, 2013). However, evidence from recent systematic reviews reveals a varied picture regarding their effectiveness. For example, while meta-analysis suggests that programmes can be effective in improving clinical symptomology in adults (Tol *et al.* 2011; Morina *et al.* 2017) much less is known about the impact of similar interventions on psychosocial outcomes such as resilience and hope (Bangpan *et al.* 2017). When delivered to children, the extent to which programmes achieve their intended impacts is inconsistent when comparing evidence across MHPSS outcomes and humanitarian contexts (Tol *et al.* 2011; Jordans *et al.*, 2016; Bangpan *et al.* 2017). Further, no clear pattern in the data has been identified to suggest which participant characteristics or whether wider social factors moderate the effect of MHPSS programmes (Bangpan *et al.* 2017; Brown *et al.* 2017). These findings highlight the need to explore contextual factors influencing the delivery and engagement in MHPSS programmes to contribute to our understanding of heterogeneity in treatment effects (Tol *et al.* 2013). For example, access to local and national resources, availability of organisational support with community partners (O'Hanlon & Budosan, 2015) and the integration of MHPPS programmes with local health and social care systems can support or hinder programme fidelity and impact (WHO, 2015; Van Ommeren *et al.* 2015).

Notwithstanding the recommendations on MHPSS by the Inter-Agency Standing Committee, which advocates taking a wide range of possible responses to humanitarian emergencies, many empirically evaluated MHPSS programmes continue to draw from Western-based approaches to the treatment of trauma and trauma-related symptoms (Bangpan *et al.* 2017). Yet, the majority of humanitarian emergencies occur in non-Western, low-resource settings where such approaches may not be feasible or applicable, (Attanayake *et al.* 2009; Chowdhary *et al.* 2014). While MHPSS programmes often take a broader non-trauma based approach, which seeks to strengthen protective factors by focusing on resilience and by enhancing a sense of empowerment, social connectedness and other family, community and economic supports (Wessells, 2009; Shah, 2012; Somasundaram, 2014), they continue to remain underrepresented in the outcome evaluation literature (Tol *et al.* 2011). To improve our understanding of the full range of MHPSS provision and approaches taken across different emergency settings further empirical investigation would benefit from engaging with programme complexity, theories of change and diversity in outcome measurements and implementation processes (Craig *et al.* 2008; Petticrew, 2015). This could also provide vital insights into how MHPSS programmes can be better integrated into existing healthcare systems and services (Ventevogel *et al.* 2011*b*). The views and perspectives of programme recipients and providers play an important role in developing a more comprehensive MHPSS evidence base, as they can provide key insights regarding important contextual and process factors, both in relation to understanding the barriers faced during implementation and how recipient characteristics and experiences of programmes influence uptake and engagement. To our knowledge, there is a lack of systematic reviews synthesising evidence from studies of people's experiences and perspective of MHPSS programmes (Bangpan *et al.* 2016). To address this gap, we report findings from our qualitative evidence synthesis on the barriers to, and facilitators of, implementing and receiving MHPSS programmes delivered to populations affected by humanitarian emergencies in low- and middle-income countries (LMICs). Qualitative data can support a greater understanding, from the perspectives of key stakeholders, what factors need to be taken into consideration to ensure programme feasibility, acceptability and uptake to inform the design and delivery of future MHPSS programmes.

## Methods

This systematic review was described *a priori* in a research protocol (Bangpan *et al.* 2016) and adheres to the PRISMA (Preferred Reporting Items for Systematic Reviews and Meta-Analyses) guidance found in the Supplementary material (Moher *et al.* 2009).

### Inclusion criteria

Studies published in English from 1980 onwards were included if they contained qualitative evidence on the perspectives of adults or children who had engaged in, or programmes providers involved in delivering, MHPSS programmes for populations affected by humanitarian emergencies in LMICs. For this review, humanitarian emergencies were defined as any natural or man-made emergencies, including both slow-onset and sudden crises. We adopted the Inter-Agency Standing Committee's (IASC) definition of MHPSS and included any programme seeking ‘to protect or promote psychosocial well-being and/or prevent or treat mental disorder’ (IASC, 2007, p. 11).

### Search

We conducted a comprehensive search of 12 electronic databases covering health and social science disciplines including Medline, PsycINFO and ASSIA, 13 specialist databases and grey literature portals, in addition to 25 topic-specific websites, consultation with experts and citation checking of includes. (See further details in the web appendix). Key index and free text search terms were determined by the review questions and the inclusion criteria. For example, the type of humanitarian emergency (e.g. war or typhoon or genocide), combined with the type of mental health and psychosocial intervention (e.g. CBT, NET) and study design (e.g. ethnography, process evaluation).

Search results were imported into EPPI-Reviewer 4: systematic review software (Thomas *et al.* 2010). References were initially screened on title and abstract. Full reports were obtained for those references where title and abstract suggested the study was relevant or where there was insufficient information to judge. We piloted the inclusion criteria by comparing the decisions of two reviewers before moving to screening by a single reviewer, any differences were resolved through discussion.

### Data extraction and quality appraisal

Data were extracted from studies using tools developed specifically for this review. Key information included: bibliographic details, participant and intervention characteristics study methods and findings (see web appendix). Piloting and refinement of tools took place before the commencement of full coding. The reliability and usefulness of studies were assessed using EPPI-Centre tools for qualitative studies using the following dimensions: sampling, data collection, data analysis, the extent to which the study findings were grounded in the data (criteria 1–4) and; the extent to which the study privileged the perspectives of participants, and breadth and depth of findings (criteria 5–6). An overall judgement of study quality was made according to two key dimensions. First, a weight of high, medium or low was assigned according to the reliability of the study using criteria 1–4. Second, a weight of high, medium or low was assigned according to the usefulness of the findings in answering the review question on contexts and barriers to implementation and receipt of MHPSS programmes using criteria 5–6. To be judged as ‘high’ quality on methodological reliability, studies needed to have taken steps to ensure rigour in at least three of the first four criteria. Studies were judged as ‘medium’ when scoring on only 2–3 criteria and ‘low’ when scoring on only one or none. To achieve a rating of high on usefulness in answering the review questions, studies needed to achieve depth and breadth in their findings and use methods that enabled participants to express their views on implementing or engaging in programmes. Studies rated as a medium on usefulness only met either one of these criteria and studies rated low were judged to have met neither criterion. Low-quality studies were not excluded from the review. Instead, quality judgements were used to inform the synthesis with none of the themes solely generated by studies judged as low on both dimensions (reliability of usefulness.

### Data synthesis

The data contained in studies in the form of participants’ quotes, authors’ descriptions or authors’ conclusions, was extracted and coded by two reviewers. The authors read and re-read the data contained within each study. They applied line-by-line codes to capture and interpret the meaning of data and organised the coding of that data into themes and higher-order themes. They met to discuss their individual coding before agreeing on a final set of themes. A process of interpretation also led to a discussion on whether there was sufficient data to inform a sub-theme, and the identification of negative case examples. Evidence tables were prepared to facilitate the writing of the narrative thematic synthesis of the findings. These tables contained the methodological quality of each study; contextual details of the programmes and humanitarian settings examined; details about the population; and the final set of themes.

## Results

### Search results

**Fig. 1.**
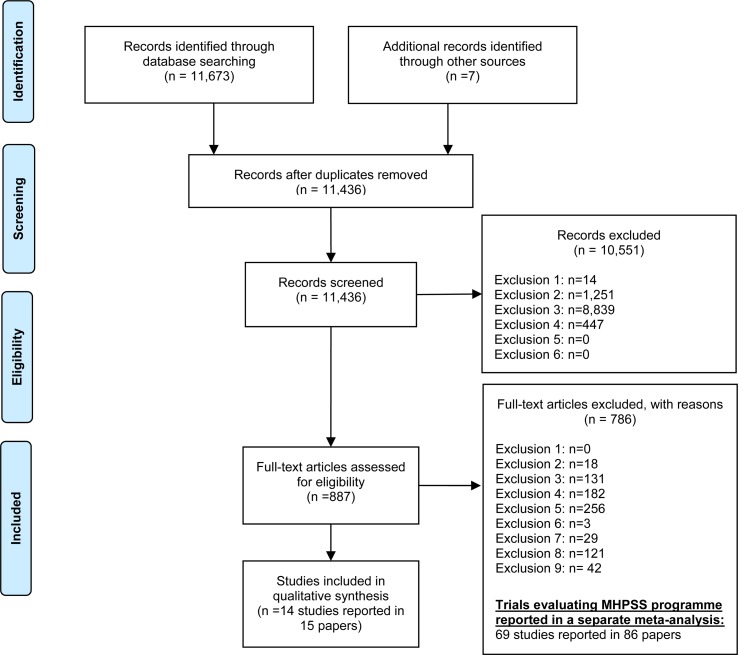
PRISMA flow diagram: The study identification, screening and selection process

The flow of studies through the review is provided in Fig. 1. After the removal of 242 duplicates, 11437 studies were identified from the search. Fourteen distinct studies met the inclusion criteria. An overview of the studies included in the review is provided in Table 1.

**Table 1.** Description of included studies

		Details of the setting and programme			Details of the study	
IASC tiers	Study	Humanitarian crises	Intervention	Programme timing	Study population	Methods of evaluation
*Tier 4: Specialised services N* = 3)	Baingana and Onyango (2011)	Civil war: Northern Uganda; until 2006	*Primary mental health* and community outreach service for adults and children	*Post-conflict:*	*Providers: n* = not stated *Recipients: n* = not stated	*Qualitative:* Document review; Key informant interviews; Patient focus groups; Clinical field observations
	Budosan & Bruno (2011)	Earthquake: Haiti; 2010	Various models of *specialised MHPSS programmes* for adults affected by the 2010 earthquake in Haiti	*Immediately after crisis*	*Providers: n* = not stated *Recipients: n* = not stated	*Qualitative:* Document review; Semi-structured interviews; Patient focus groups
	Shackman & Price (2013)	Civil war: Sierra Leone; 1991–2002	*Primary mental health* and community outreach service for adults and children	*Post-conflict*	*‘Key stakeholders’^a^: N* = 206	*Qualitative*: Document review; Individual and focus group interviews
	Song *et al.* (2013)	Civil war: Sierra Leone; 1991–2002	*Primary mental healthcare:* psychiatric hospitals and community mental health services for former child soldiers	*Post-conflict*	*Providers: n* = 24	*Qualitative:* Semi-structured interviews
*Tier 3: Focused, non-specialised support N* = *3*)	Chauvin *et al.* (1998)	Genocide: Rwanda; 1994–1995	*Psychosocial trauma recovery programme* for children and care-givers in Rwanda	*Immediately after conflict*	*Providers: n* = not stated	*Qualitative* Document review; Semi-structured interviews; Patient focus groups; Clinical field observations
	Hogwood *et al.* (2014)	Genocide: Rwanda; 1994–1995	*Community counselling groups* for adult women in Rwanda	*Post-conflict*	*Recipients: n* = 40	*Qualitative/Quantitative:* Rating scale Focus groups
	King (2014)	Genocide: Rwanda; 1994–1995	*The Healing of Life Wounds programme* for adults in Rwanda	*Post-conflict*	*Recipients: n* = 23	*Qualitative/Quantitative:* Rating scales, Focus groups
*Tier 2: Community and family support N* = 6)	Boothby *et al.* (2006)	Civil war: Mozambique; 1977–1992	*Children and war rehabilitation psychological and social programme* for former male child soldiers	*Ongoing to post-conflict:*	*Recipients*: *n* = 23	*Qualitative:* Interviews; Focus groups
	Kunz (2009)	Earthquake: Iran; December 2003	*Sport and play programme* for children and youth (6–18 years old)	*Post-conflict*	*Providers: n* = not stated *Recipient parents: n* = 15	*Qualitative/Quantitative:* Semi-structured interviews; Questionnaires
	Lykes & Crosby (2014)	Guatemala: armed conflict	*Creative arts project* with Mayan women in Guatemala	*Post-conflict*	*Recipients: n* = 105	*Qualitative:* Participatory research workshops
	Nakkash et al. (2012)	Palestinian/Israeli conflict; ongoing	Qaderoon’ (We Are Capable) *social skills building programme* for refugee children (11–14 years old) in Palestine	*Ongoing*	*Providers: n* = not stated *Recipients: n* = 150	*Qualitative/Quantitative:* Interviews; Clinical field observations forms; Recipient satisfaction form
	Nastasi *et al.* (2011)	Tsunami: Sri Lanka; December 2004	*Sri Lanka:* post-tsunami *after-school programme* for students	*Post-crisis*	*Providers: n* = not stated *Recipients: n* = 120	*Qualitative/Quantitative*; Curriculum feedback activities, Student evaluation forms; Teacher evaluation forms
	Sahin et al. (2011)	Earthquake: Marmara, Turkey; 1999	*School-based psycho-educational programme* for children and parents after the 1999 earthquake in Turkey	*Immediately after crisis*	*Recipients:* Children: 593 Parents *n* = 137	*Qualitative/Quantitative:* Questionnaires
*Tier 1: Basic services and security (N* = *1)*	Christensen & Edward (2015)	Civil war: Burundi; 1972 and 1994	*Village health worker clinic* integrating health delivery with other community development initiatives for the community in Burundi	*Post-conflict*	*Providers: n* = 37 *Recipients: n* = 80	*Qualitative* Open-ended interviews, Focus groups

a Breakdown by providers and recipients not provided.

### Characteristics and quality of included studies

We mapped programmes against the tiered system of complementary MHPSS supports, developed by the Inter-Agency Standing Committee (IASC) reference group responsible for the greater co-ordination of MHPSS in emergency settings (IASC, 2007). We found the evidence base included tier-four ‘specialised services’ delivered in post-conflict settings (*n* = 3) and immediately after an earthquake (*n* = 1), tier-three ‘focused, non-specialised supports’ programmes addressing the psychological and social impact of the Rwandan genocide (*n* = 3); while tier-two ‘community and family supports’ programmes primarily targeted children (*n* = 5) rather than adults (*N* = 1). Only one study addressed the basic services and security needs of affected populations. Overall, study quality was a combination of high or medium reliability and usefulness (*n* = 10). Of the four studies judged as being of low reliability, three contributed findings of medium usefulness (see Table 2).

**Table 2.** Reliability and usefulness of findings

Study	Reliability			Usefulness		
	High	Medium	Low	High	Medium	Low
Baingana & Onyango (2011)			✓		✓	
Boothby *et al.* (2006)		✓		✓		
Budosan & Bruno (2011)			✓		✓	
Chauvin *et al.* (1998)			✓			✓
Christensen & Edward (2015)	✓			✓		
Hogwood *et al.* (2014)		✓		✓		
King (2014)		✓		✓		
Kunz (2009)		✓		✓		
Lykes & Crosby (2014)		✓		✓		
Nakkash et al. (2012)	✓				✓	
Nastasi *et al.* (2011)	✓				✓	
Sahin *et al*. (2011)	✓				✓	
Shackman & Price (2013)			✓		✓	
Song *et al.* (2013)	✓			✓		

### Thematic synthesis

#### Theme 1: Engagement with local communities and government agencies

A key theme across nine studies (Chauvin *et al.* 1998; Boothby *et al.* 2006; Kunz, 2009; Baingana & Onyango, 2011; Budosan & Bruno, 2011; Nakkash *et al.* 2012; Shackman & Price, 2013; Song *et al.* 2013; Christensen & Edward, 2015), was the importance of formal and informal engagement with local communities and government agencies to support the implementation and coordination of MHPSS programmes in humanitarian settings. Subthemes included the importance of community mobilisation and sensitisation, establishing good relationships with parents and developing effective local community and government partnerships.

### Community mobilisation and sensitisation

Sensitising and mobilising communities about the potential impact of humanitarian emergencies were cited as key programme activities in five studies. Two of which were judged to be highly useful and of high (Christensen & Edward, 2015) or medium reliability (Boothby *et al.* 2006). Two lower-quality studies also provided medium useful findings, (Baingana & Onyango, 2011; Shackman & Price, 2013) while one study was judged as being of low reliability and usefulness (Chauvin *et al.* 1998). Programme providers identified the need to increase knowledge about the effects of extended periods of exposure to violence and conflict on psychosocial outcomes. For example, to mitigate the impact of child soldiering experiences on young boys in Mozambique, Boothby *et al.* (2006) found that their tier-two programme rehabilitation activities needed not only to focus on psychological recovery but also on the reintegration of former child soldiers (FCSs) into their home communities. This was achieved via ‘community sensitisation campaigns’ focused on increasing ‘community acceptance’ of FCSs. Programmes targeted public services to encourage ‘collective responsibility’ to ‘support the reintegration of FCS’ (p. 97). Similarly, an evaluation of a tier-one integrated village health clinic found that mobilising and sensitising the community in post-conflict Burundi instilled a previously missing ‘sense of purpose’ (Christensen & Edward, 2015, p. 48). Increasing access to tier-four community-based primary mental healthcare by reducing stigma towards people with mental health problems was also identified in two studies. In post-conflict Northern Uganda, Baingana & Onyango (2011) found that community mobilisation and sensitisation had been ‘effectively carried out’ in the region, leading to ‘exceeding targets’ of 120 to closer to 200 patients engaging with mental health clinics. In post-conflict Sierra Leone, Shackman & Price (2013) found that ‘radio programmes were the most successful outreach activities in terms of reaching out and disseminating information to community members’ with recipients stating that ‘negative attitudes towards them changed for the better after they had received support and had recovered’ (p. 269). Chauvin *et al.* (1998), reporting on a tier-three psychosocial trauma recovery programme for children in Rwanda, supported these findings. They also advocated strengthening community sensitisation activities via a ‘mass media campaign’ and liaising with individuals delivering community services to increase programme reach (p. 390).

### Establishing good relationships with parents to support uptake of MHPSS programmes

Establishing the engagement and trust of parents could be a challenge but was seen as essential in ensuring uptake of services targeting children and young people, a sub-theme identified in two studies evaluating tier-two community and family support programmes in a post-conflict and post-disaster setting. For example, the study by Nakkash *et al.* (2012) judged to be of high reliability and medium usefulness, found that although children were ‘eager to attend’ a weekly social skills building programme, parents were often a ‘barrier’ (p. 602) as they placed greater value on their children's education, rather than recreational activities. Programme providers took steps to address this issue by incorporating English reading sessions to ‘increase the perceived educational value of the program’ (p. 602). Developing good channels of communication with parents was also highly valued in the evaluation of a sports-based youth programme delivered to children in post-earthquake Iran by Kunz (2009), judged as providing highly useful findings of medium reliability. For example, coaches acted ‘as mediators’ to bridge any misunderstandings between children and their parents, by explaining to them that ‘weaker school performance’ may be a result of the ‘mental suffering the children had undergone during the earthquake or to their current living conditions’ and that sustained participation in sports activities, ‘could help’ young people ‘feel better and perform better’ (p. 1154).

### Developing effective local community and government partnerships

The need to improve coordination and develop effective partnerships with local community and government agencies to support greater programme implementation was identified in five studies. The quality of studies varied from highly reliable and useful (Song *et al.* 2013) to low reliability but with medium useful findings (Baingana & Onyango, 2011; Budosan & Bruno, 2011; Shackman & Price, 2013). Song *et al.*’s (2013) evaluation of a tier-four primary mental health initiative targeting FCS in Sierra Leone, reported that the delivery of effective care was hampered by a lack of coordination and communication between providers, local organisations and the government, limiting the opportunities for mutual support and sharing of learning and resources. Programme providers attributed the reluctance of their government health department to engage with and collaborate on a strategy for the coordinated delivery of mental health for affected populations to mental health ‘stigma’ and the government's lack of knowledge and skills on ‘and how to address’ mental health problems (p. 619). Shackman & Price (2013) also faced a lack of ‘government buy-in’ to support the long-term sustainability of tier-four mental health services in post-conflict Sierra Leone. An international development aid organisation also faced similar challenges with the lack of mental health prioritisation when attempting to implement a coordinated strategy for the provision of tier-four integrated MHPSS services immediately after the 2010 Haiti earthquake. They found collaboration attempts with local NGO partners in Haiti proved difficult as their focus was on the ‘development of their own human and material resources’ rather than delivery of MHPSS programmes (Budosan & Bruno, 2011, p. 233). Two further studies of low reliability supported these findings (Chauvin *et al.* 1998; Baingana & Onyango, 2011) stressing the need to strengthen coordination of MHPSS programmes at local and national levels.

#### Theme 2: Sufficient number of trained MHPSS programme providers

The importance of recruiting and retaining staff in resource-limited settings, to ensure MHPSS programmes were delivered as intended, was a concern across five studies (Chauvin *et al.* 1998; Baingana & Onyango, 2011; Budosan & Bruno, 2011; Shackman & Price, 2013; Song *et al.* 2013).

### Challenge of recruiting and retaining providers

A priority identified in four studies was ensuring that programmes were adequately staffed (Chauvin *et al.* 1998; Baingana & Onyango, 2011; Shackman & Price, 2013; Song *et al.* 2013). The highly reliable and useful study by Song *et al.* (2013) highlighted the difficulties that tier-four primary mental health services have in low-income countries, such as Sierra Leone, where they struggled to recruit ‘medical students into psychiatry’ (p. 619), citing primary disincentives, such as salaries ‘as low as $80 per month, few ancillary mental health staff in the country, and a deep-rooted stigma against mental illness’ (p. 620). The low-reliability study by Shackman & Price (2013) supported these findings. The authors reported that practitioners were often ‘poorly paid and constantly under the threat of losing their position at the end of a project cycle’ (p. 265) reflecting ‘the low priority given to mental health, the lack of resources and training’ in the field (p. 269).

In the study by Baingana & Onyango (2011) of low reliability and medium usefulness, the authors were optimistic that even with a small number of full-time staff (*n* = 8) they could ‘facilitate district health workers to establish and run mental health clinics’ (tier-four) in post-conflict Northern Uganda; however, their efforts were hindered by ‘an attrition of government health workers trained by the project’ (p. 298). Issues with staff retention meant a loss of knowledge and skills in being able to ‘recognise, assess, and manage mental illness’ (p. 298). Chauvin *et al.* (1998) a low quality study, also suggested that the ‘the number of trauma advisors need to be increased’ (p. 390) if they were to ensure effective delivery of a tier-three psychosocial trauma programme for children in Rwanda.

### Ensuring providers are sufficiently trained to deliver MHPSS programmes

Even when services were more adequately staffed, there were concerns about the extent to which providers felt sufficiently skilled to deliver and address the mental health needs of the local population in tier-four (*n* = 4) or tier-three MHPSS programmes (*n* = 1). The high-quality study by Song *et al.* (2013) reports that there was ‘lack of trained staff able to provide effective mental health and psychosocial work’ (p. 619) to assist in the rehabilitation efforts of FCSs in Sierra Leone. Three studies, judged to be of low reliability identified similar themes. For example, when attempting to deliver clinical care to adults immediately after the 2010 Haiti earthquake Budosan & Bruno (2011) found that the majority of primary healthcare practitioners felt that they ‘lacked the knowledge and skills’ (p. 230) to assist patients presenting with mental health problems. Similarly, in their evaluation of the primary mental health and community outreach services capacity to address the MHPSS needs of people affected by the civil war in Uganda, Baingana & Onyango (2011) found that although there were 12 members in each village health team, ‘only one was trained’ and thus it proved difficult to cover all outreach activities (p. 298) including programme components ‘specifically targeted at children’ (p. 298). Chauvin *et al.* (1998) found that although a 2-day training course included ‘sensitising’ staff to support children exposed to the Rwandan genocide, the capacity-building efforts focused primarily ‘on the human resources facet specifically for front line groups’ with more training ‘related to counselling’ needed (p. 389). Shackman & Price (2013). report that the delivery of socially and culturally appropriate tier-four mental health services, in post-conflict Sierra Leone, was hampered by training materials found to be skewed towards the ‘biomedical model’, and ‘westernised counselling techniques’ rather than supporting practitioners to identify ‘appropriate treatments’ for people in the region (p. 268). Instead, individual practitioners attempted to adapt ‘a western counselling model to better fit into their own cultural ways of helping’ (p. 268).

#### Theme 3: Experience of programme activities

Recipients across five studies evaluating tier-two MHPSS programmes (Boothby *et al.* 2006; Kunz, 2009; Nastasi *et al.* 2011; Sahin *et al.* 2011; Lykes & Crosby, 2014) provided diverging views on their experiences of engaging in programme activities.

### Increasing meaningful and enjoyable engagement through the provision of varied and creative activities

The extent to which engagement in MHPSS programmes was more enjoyable or meaningful to recipients when they included a range of activities, including creative or other forms of play was identified across three studies. Two studies were judged as highly reliable and providing medium useful findings (Nastasi *et al.* 2011; Sahin *et al.* 2011) and one study of medium reliability with highly useful findings (Lykes & Crosby, 2014).

Sahin's *et al.* (2011) evaluation of a psycho-education programme on the impact of natural disasters for children and adults after the Marmara earthquake, indicated that for parents increasing the number of issues covered and made available for group discussion increased the perceived benefits. For children, the perceived benefits also increased when ‘the number and variety of activities’ was higher (p. 46). Data from student evaluation forms revealed mixed findings about the content of an after-school psychosocial curriculum, delivered in post-tsunami Sri Lanka, with some young people ‘indicating enjoyment of writing, drawing, working together, and questioning, while others indicated dislike’ for the same activities (Nastasi *et al.* 2011, p. 527). The study by Kunz (2009), found that many of the young people sampled ‘looked forward’ to engaging in sports and play activities, not only because they were ‘fun’ but because participating in the programme was ‘very important’ to them. When evaluating ‘creativity as an intervention strategy’ (p. 30) for Mayan women living in post-conflict Guatemala, Lykes & Crosby (2014) found that the inclusion of activities such as drawing and drama enabled women to engage more meaningfully in the healing process. The women spoke about the value of embodying their experiences via performances, helping them bypass words to express emotional effects, such as ‘sadness, negative memories, suffering that we have lived through’, as well as enabling them to ‘enjoy things’ again (p. 38).

### Culturally relevant activities

A further sub-theme, identified in two highly useful and medium reliable studies was the importance of culturally relevant activities to support engagement and increase programme impact. Interviews with FCSs in the study by Boothby *et al.* (2006) found that participation in traditional cleansing ceremonies ‘helped them return to civilian life’ and were ‘vital for rebuilding’ trust between the boys and their communities’ (p. 96). Similarly, Lykes & Crosby (2014) reported that to ensure programme effectiveness and support deeper forms of engagement, programme activities needed to be in alignment with the ‘cultural and educational’ experience of Mayan women. By developing a creative-based workshop that ‘interfaces with Mayan beliefs and practices’, providers were able to support ‘indigenous meaning making’ and facilitate women's engagement ‘in a wide range of processes that contributed to their personal transformation’ (p. 38).

#### Theme 4: Benefits of group-based programmes

The benefits of group-based MHPSS programmes were cited in five studies (Nastasi *et al.* 2011; Hogwood *et al.* 2014; King, 2014; Lykes & Crosby, 2014; Christensen & Edward, 2015).

### A resource and source of support

A sub-theme in four studies was the importance of the group as a resource and a source of support. Two studies were judged as highly reliable and provided high (Christensen & Edward, 2015) and medium useful findings (Nastasi *et al.* 2011), the remaining two studies were of medium reliability both providing highly useful findings (Hogwood *et al.* 2014; Lykes & Crosby, 2014). Community members in the evaluation of a tier-one health clinic by Christensen & Edward (2015) valued ‘being drawn together as a group by enjoyable things’ (p. 40). The role of group-based programmes in promoting social cohesion and ‘reducing social isolation’, by connecting with others in a similar situation, was also reported by women participants in a tier-three counselling support group for mothers exposed to events during the Rwandan genocide (Hogwood *et al.* 2014). The study found that women ‘attributed their improved relationship with their children to the support and knowledge provided by the group’ (Hogwood *et al.* 2014, p. 401). Two studies evaluating tier-two community and family support interventions also reported favourable experiences of participants engaged in group-based activities. In Nastasi *et al.*’s (2011) evaluation of a post-tsunami after-school programme delivered to young people in Sri Lanka, the findings revealed that ‘the opportunity for group interaction’ was met positively and corresponded with ‘high levels of engagement’ by students (p. 527). Lykes & Crosby (2014) also found that Mayan women in post-conflict Guatemala who were engaged in collective drawing activities valued ‘the many opportunities of doing things together’ (p. 36).

### Safe space to tell their story

Two studies, evaluating tier-three programmes for women in post-genocide Rwanda, provided highly useful evidence of medium reliability on the challenging but rewarding experience of sharing their personal stories with others. In the Healing of Life Wounds programme, King (2014) found that many of the women appreciated the confidentiality of the group as a separate and ‘safe space’ from the wider community, where they could process their emotions. The study reported that forming trust among women in the group was a gradual process that evolved over time as more women shared their experiences and felt heard and listened to with respect and empathy. In her study of community counselling groups for Rwandan mothers, Hogwood *et al.* (2014) reported that, although some group members found it difficult to talk about their experiences, ultimately, realising that they were not alone helped them to find their voice.

#### Theme 5: Quality and nature of relationships with programme providers

A key theme across four studies (Boothby *et al.* 2006; Kunz, 2009; King, 2014; Lykes & Crosby, 2014) was the quality and nature of relationships between providers and recipients, and their role in maximising engagement and increasing the impact of programmes.

### Building trusting and supportive relationships

The importance of building trusting and supporting relationships was a sub-theme emerging from two studies judged to be of medium reliability and providing highly useful findings. In the post-earthquake city of Bam, Kunz (2009) evaluation of a sports programme for young Iranian people found that once ‘a trusted relationship’ between the sports coaches and young people was established, it was likely to be ‘an indicator for positive development’ (p. 1153). Similarly, the trust and support received by facilitators in a creative arts project for Mayan women in post-conflict Guatemala were reflected on positively. Women spoke about ‘coming out of our fear’ to process their experiences as being a direct result of the facilitators working with them (Lykes & Crosby, 2014).

### Personal qualities and providers acting as role models

In addition to the importance of building trusting and supportive relationships, programme recipients in three studies, providing highly useful findings of medium quality, also reflected on the individual qualities and attributes of programme providers, citing them as key factors in supporting them to participate and benefit from MHPSS programmes. For example, in the evaluation of a tier-three programme by King (2014) adult survivors of the Rwandan genocide spoke emphatically about the professional qualities of the primary facilitator. They described him as someone who was ‘calm, humble, attentive and compassionate’, with the ability to ‘handle crises; welcome opposing views without taking sides; be flexible and disclose his own personal challenges despite his social status and age’ (p. 423). These skills were seen as pivotal in facilitating and bringing together Tutsis and Hutus to engage in the Healing of Life Wounds dialogue and reconciliation programme. The study by Boothby *et al.* (2006) reported similar findings by FCSs engaged in the tier-two Mozambique-based Children and War Rehabilitation psychological and social programme. Young people in this study appreciated the care-givers’ concern for their ‘well-being, including appropriate discipline, and consistent modelling of good behaviour’ (p. 99). They credited these qualities with helping ‘them to recover their own sense of caring for other human beings’ (p. 99). The young people in the tier-two psychosocial sports programme in post-earthquake Bam in Iran also valued the coaches’ qualities of ‘understanding and caring about people’ over their being ‘good in sports’ (Kunz, 2009, p. 1153). The establishment of a supportive relationship based on these qualities enabled coaches to also ‘serve as role models for the children’ and provided them ‘with guidance and orientation’ (p. 1154).

## Discussion

A number of themes emerged from our synthesis. Community engagement was identified as a key mechanism to support the successful delivery and uptake of MHPSS programmes in humanitarian settings. In particular, mental health sensitisation and mobilisation strategies, and the need to develop effective partnerships with governments and local communities were seen as pivotal to increasing overall programme accessibility and reach. These findings resonate with a growing body of literature on contextual factors influencing effective implementation of MHPSS programmes in humanitarian settings (Kruk *et al.* 2010; Tol *et al.* 2011; Tol *et al.* 2013). For example, collaborating with governments to deliver services, co-ordination of multi-sectorial mental health agencies to maximise resources and reduce potential duplication of effort and investing in the provision of integrated mental health and psychosocial activities with existing primary healthcare have all emerged as critical to addressing the short and long-term MHPSS needs of affected communities (Pérez-Sales *et al.* 2011; Ventevogel *et al.* 2011*a*, *b*; Epping-Jordan *et al.* 2015). Successful collaboration efforts, to enable sustainable programme development, also require sensitive assessment of local political conditions and current views on service provision, and the time and commitment needed to invest in relationships and effective partnerships (Patel *et al.* 2011).

Another key mechanism contributing to the successful implementation of MHPSS programmes is ensuring they are delivered by sufficient numbers of trained providers. However, the recruitment and retention of practitioners sufficiently skilled to deliver MHPSS programmes continues to be an ongoing challenge, especially in resource-limited settings where there may be a lack of incentives to work in the mental health sector (Eisenbruch *et al.* 2004; Budosan & Aziz, 2009; Budosan & Bruno, 2011). These findings underscore the need to explore the potential of different training approaches such as the ‘apprenticeship model’ and ‘training of trainers’ to ascertain if they can support organisations to develop and retain practitioners at the level of skill and numbers required to ensure programme fidelity and reach (Murray *et al.* 2011; Quosh, 2013; Budosan *et al.* 2014; Henderson *et al.* 2016).

Another key theme was the importance of designing programmes that are socially and culturally meaningful to local populations to ensure they are appealing and achieve their intended aims. The importance of attending to cultural and ethical issues when supporting the mental health and psychosocial well-being of different groups is well documented (Chu *et al.* 2016) and is one of the key challenges to the delivery of MHPSS programmes in diverse cultural settings in which emergencies often occur (Sommers-Flanagan, 2007; Wessells, 2009). To address this challenge, development of MHPSS programmes should consider exploring the needs of local communities by drawing on participatory approaches to facilitate stakeholder involvement in the design and delivery of programmes (Oliver *et al.* 2008; Mockford *et al.* 2011). The findings also highlight the importance of ensuring that programmes remain appealing and accessible to local populations by increasing opportunities for meaningful engagement via peer group support. Engagement with peers in group-based programmes was seen as beneficial as it provided an opportunity to connect with people from similar circumstances and backgrounds, helping to promote greater social cohesion and reduce social isolation. An evaluation of psychosocial activities for children living in armed conflict settings also found high levels of satisfaction with group-based programmes (Jordans *et al.* 2011). This finding was moderately associated with a reduction in post-treatment problems, reflecting increasing evidence on the relationship between participant satisfaction and overall programme effectiveness (Mueller & Pekarik, 2000; Roos & Werbart, 2013).

A final theme in our synthesis concerned the importance of building trusting and supporting relationships between programme providers and recipients to maximise engagement and increase programme impact. We found that providers who could relate by bridging differences and show nurturing qualities, and who could act as role models, were highly valued. The wider research literature also attests to the value of establishing a robust and attuned therapeutic relationship to improve recipient outcomes (Norcross & Wampold, 2011). In humanitarian settings, attention therefore needs to be given to the individual qualities of programme providers and to the potential to add value by investing in high-quality relationships to improve people's mental health and psychosocial well-being.

### Strengths and limitations

We have taken a systematic and transparent approach to synthesising qualitative evidence on the implementation and receipt of MHPSS programmes delivered to populations affected by humanitarian emergencies in LMICs; filling a notable gap in the evidence base. Although our search was successful in locating qualitative studies, their methodological reliability and usefulness varied. Some studies lacked analytical depth, and thus important themes may have been missed. The relatively smaller number of studies on the impact of natural disasters and the predominance of findings from post-conflict settings may also have obscured findings specifically relevant to those settings. Despite conducting a comprehensive and sensitive search, we cannot be certain that we found all relevant studies; in particular, we may not have identified grey literature or unpublished reports, which are more likely to include qualitative evidence on the process of implementing programmes. Limiting the review to English language studies also means key insights from other languages have not been included. However, despite these limitations, we developed a contextually rich synthesis exploring contextual factors that can be taken into consideration to facilitate greater programme feasibility, fidelity, acceptability and reach.

## Conclusions

This review has synthesised qualitative evidence from process evaluations on the implementation and receipt of MHPSS programmes delivered to people affected by humanitarian emergencies. Our findings suggest that future MHPSS programmes and services would benefit from continuing to invest in community engagement and outreach efforts to promote the value and improve the co-ordination of MHPSS services at local and national levels; explore models of best practice to support the training needs of providers, deliver culturally sensitive and socially appropriate MHPSS programmes for individuals and their families and build high quality therapeutic relationships to improve recipient engagement and outcomes. Further research should build on these findings and current practice recommendations, alongside evaluations of programme effectiveness. This could support better theorisation on the links between programme aims, choice of programme components, delivery mechanisms and how programmes intend to improve outcomes for affected populations.

## Supplementary material

For supplementary material accompanying this paper visit http://dx.doi.org/10.1017/gmh.2018.12. click here to view supplementary material
