# Supplementary material for: What are the barriers to, and facilitators of, implementing and receiving MHPSS programmes delivered to populations affected by humanitarian emergencies? A qualitative evidence synthesis
Source: Glob Ment Health (Camb). 2018 Jun 1;5:e21. doi: 10.1017/gmh.2018.12 (PMC6036649; doi:10.1017/gmh.2018.12)
Supplement: Supplementary file 1 [file S2054425118000122sup001.docx]

**Web Appendix**

# **PRISMA checklist**

| Section/topic | # | Checklist item | Reported on page # |
| --- | --- | --- | --- |
| TITLE | | |  |
| Title | 1 | Identify the report as a systematic review, meta-analysis, or both. | #1 |
| ABSTRACT | | |  |
| Structured summary | 2 | Provide a structured summary including, as applicable: background; objectives; data sources; study eligibility criteria, participants, and interventions; study appraisal and synthesis methods; results; limitations; conclusions and implications of key findings; systematic review registration number. | Abstract |
| INTRODUCTION | | |  |
| Rationale | 3 | Describe the rationale for the review in the context of what is already known. | #1 |
| Objectives | 4 | Provide an explicit statement of questions being addressed with reference to participants, interventions, comparisons, outcomes, and study design (PICOS). | #2 |
| METHODS | | |  |
| Protocol and registration | 5 | Indicate if a review protocol exists, if and where it can be accessed (e.g., Web address), and, if available, provide registration information including registration number. | #2 |
| Eligibility criteria | 6 | Specify study characteristics (e.g., PICOS, length of follow-up) and report characteristics (e.g., years considered, language, publication status) used as criteria for eligibility, giving rationale. | #3 |
| Information sources | 7 | Describe all information sources (e.g., databases with dates of coverage, contact with study authors to identify additional studies) in the search and date last searched. | #3 and further detail in web appendix |
| Search | 8 | Present full electronic search strategy for at least one database, including any limits used, such that it could be repeated. | Can provide on request |
| Study selection | 9 | State the process for selecting studies (i.e., screening, eligibility, included in systematic review, and, if applicable, included in the meta-analysis). | #4 |
| Data collection process | 10 | Describe method of data extraction from reports (e.g., piloted forms, independently, in duplicate) and any processes for obtaining and confirming data from investigators. | #4 |
| Data items | 11 | List and define all variables for which data were sought (e.g., PICOS, funding sources) and any assumptions and simplifications made. | #3 |
| Risk of bias in individual studies | 12 | Describe methods used for assessing risk of bias of individual studies (including specification of whether this was done at the study or outcome level), and how this information is to be used in any data synthesis. | Quality appraisal provided #6 |
| Summary measures | 13 | State the principal summary measures (e.g., risk ratio, difference in means). | n/a |
| Synthesis of results | 14 | Describe the methods of handling data and combining results of studies, if done, including measures of consistency (e.g., I^2^) for each meta-analysis. | Equivalent for qualitative synthesis: #5 |
| Section/topic | # | Checklist item | Reported on page # |
| Risk of bias across studies | 15 | Specify any assessment of risk of bias that may affect the cumulative evidence (e.g., publication bias, selective reporting within studies). | Quality appraisal provided #6 |
| Additional analyses | 16 | Describe methods of additional analyses (e.g., sensitivity or subgroup analyses, meta-regression), if done, indicating which were pre-specified. | n/a |
| RESULTS | | |  |
| Study selection | 17 | Give numbers of studies screened, assessed for eligibility, and included in the review, with reasons for exclusions at each stage, ideally with a flow diagram. | #5-6 (figure 1) |
| Study characteristics | 18 | For each study, present characteristics for which data were extracted (e.g., study size, PICOS, follow-up period) and provide the citations. | #6 and table 1 |
| Risk of bias within studies | 19 | Present data on risk of bias of each study and, if available, any outcome level assessment (see item 12). | Quality assessment: Table 2: Reliability and usefulness of findings |
| Results of individual studies | 20 | For all outcomes considered (benefits or harms), present, for each study: (a) simple summary data for each intervention group (b) effect estimates and confidence intervals, ideally with a forest plot. | n/a |
| Synthesis of results | 21 | Present results of each meta-analysis done, including confidence intervals and measures of consistency. | Meta-synthesis:  #7-24 |
| Risk of bias across studies | 22 | Present results of any assessment of risk of bias across studies (see Item 15). | Table 2: Reliability and usefulness of findings |
| Additional analysis | 23 | Give results of additional analyses, if done (e.g., sensitivity or subgroup analyses, meta-regression [see Item 16]). | n/a |
| DISCUSSION | | |  |
| Summary of evidence | 24 | Summarize the main findings including the strength of evidence for each main outcome; consider their relevance to key groups (e.g., healthcare providers, users, and policy makers). | #25 |
| Limitations | 25 | Discuss limitations at study and outcome level (e.g., risk of bias), and at review-level (e.g., incomplete retrieval of identified research, reporting bias). | #24 |
| Conclusions | 26 | Provide a general interpretation of the results in the context of other evidence, and implications for future research. | #26-2 |
| FUNDING | | |  |
| Funding | 27 | Describe sources of funding for the systematic review and other support (e.g., supply of data); role of funders for the systematic review. | Supplied on request of submission |

# **Methods**

## Search strategy

#### 2.2.1 Sources

#### Key informants

We contacted advisory group members and a network of our review team’s topic expert (AC) to identify relevant unpublished literature. This was particularly important for identifying ‘grey literature’ that is often not in the public domain.

#### Electronic databases

The following 12 bibliographic databases, across disciplines, were searched: Medline, ERIC, PsycINFO, Econlit, Cochrane Library, IDEAS, IBSS, Sociological Abstracts, Social Sciences Citation Index (SSCI), CINAHL, Embase, Scopus and ASSIA. Special list databases and grey literature were also searched: Global Health Library, Health Management Information Consortium (HMIC), POPLINE, British Library for Development Studies, DFID [(http://r4d.dfid.gov.uk/)](http://r4d.dfid.gov.uk/), International Initiative for Impact Evaluation (3ie), ELDIS, greylit.org, Google Scholar, PROSPERO, WHO International Clinical Trials Registry Platform (ICTRP), ISCTRN and ClinicalTrials.gov.

#### Websites

- The World Bank:<http://www.worldbank.org/>
- The Overseas Development Institute (ODI), including the Humanitarian Policy Group: <http://www.odi.org/programmes/humanitarian-policy-group>(HPG) and Humanitarian Practice Network:<http://odihpn.org/>(HPN)
- Institute of Development Studies:<http://www.ids.ac.uk/>
- International Development Research Centre:<http://www.idrc.ca/EN/Pages/default.aspx>
- Active Learning Network for Accountability and Performance in Humanitarian Action (ALNAP):<http://www.alnap.org/>
- Emergency Nutrition Network (Field Exchange):<http://www.ennonline.net/>
- Evidence Aid:<http://www.evidenceaid.org/>
- Feinstein International Center, Tufts University:<http://fic.tufts.edu/>
- Enhanced Learning and Research for Humanitarian Assistance:<http://www.elrha.org/>
- International Association of Professionals in Humanitarian Assistance and Protection: <https://phap.org/>
- Humanitarian Accountability Partnership:<http://www.hapinternational.org/>(now CHS Alliance)
- Network on Humanitarian Action:<http://nohanet.org/>
- Harvard Humanitarian Initiative:<http://hhi.harvard.edu/>
- Refugee Studies Centre, University of Oxford:<http://www.rsc.ox.ac.uk/>
- European Commission Humanitarian Aid and Civil Protection Department (ECHO): <http://ec.europa.eu/echo/>
- USAID Development Experience Clearinghouse (and related USAID sub-websites):
- <https://dec.usaid.gov/dec/home/Default.aspx>
- ReliefWeb:<http://reliefweb.int/>
- Oxfam Policy and Practice:<http://policy-practice.oxfam.org.uk/>
- Mental Health and Psychosocial Support Network:<http://mhpss.net/>
- UNHCR:<http://www.unhcr.org/cgi-bin/texis/vtx/home>
- UNICEF:<http://www.unicef.org.uk/>
- Asian Development Bank:<http://www.adb.org/about/main>
- African Development Bank:<http://www.afdb.org/en/>
- Inter-American Development Bank: [http://www.iadb.org/en/inter-american-developmentbank,2837.html](http://www.iadb.org/en/inter-american-development-bank,2837.html)
- United Nations Office for the Coordination of Humanitarian Affairs (OCHA): <http://www.unocha.org/hina>
- International Committee of the Red Cross (ICRC):<https://www.icrc.org/en>
- Office of U.S. Foreign Disaster Assistance (OFDA), USAID: [https://www.usaid.gov/whowe-are/organization/bureaus/bureau-democracy-conflict-and-humanitarianassistance/office-us](https://www.usaid.gov/who-we-are/organization/bureaus/bureau-democracy-conflict-and-humanitarian-assistance/office-us)

#### Citation searching

We scanned citations in the reference sections of reviews and systematic reviews identified during the scoping exercise, and of studies that were subsequently included in the review, for inclusion and synthesis.

## Search strategy

Key search terms were determined by the review questions and the inclusion criteria, and were developed iteratively and piloted against papers already identified in the scoping exercise. Search strings were developed for each database, using combinations of the main key terms and their synonyms, which denoted key aspects of the review. The search used the Boolean operator ‘OR’ to link each key aspect to its synonyms; then, all key aspects were combined using ‘AND’ to identify relevant literature. Three key concepts were included in the search strings, including humanitarian, mental health and psychosocial intervention, and study design: for example, (humanitarian OR war OR conflict OR earthquake) **AND** (mental health OR psychosocial) **AND** (quantitative or effectiveness).

## CODING TOOLS: DATA EXTRACTION FOR SYNTHESIS

### Process evaluations: methodological characteristics and study findings

| **Section A: Administrative details** | |
| --- | --- |
| **Identification of report (or reports)**  *Please use as many keywords as apply* | - Citation - *Please use this keyword if the report was identified from the bibliographic list of another report.* - Contact - *Please use this keyword if the report was found through a personal/professional contact.* - Handsearch a journal - *Please use this keyword if the report was found through handsearching a journal.* - Unknown - *Please use this keyword if it is unknown how the report was found.* - Electronic database *Please specify* - Websites |
| **Type of documents**  *Please use ONE keyword only* | - Journal articles - Research reports - Programme documents (e.g. monitoring and evaluation reports) - Needs assessments - Conferences - Dissertations/thesis - Other unpublished documents |
| **Section B: Study aims and descriptive details** | |
| What are the aims of the study? | - Not stated - Details |
| What is the objective of the study? | - To evaluate the effectiveness of an intervention - To evaluate the delivery or receipt of participating in an intervention |
| When was the study conducted? (e.g. including how long after the emergency and/or the delivery of the intervention) | - Not stated - Details |
| In which country/countries was the study carried out? (please specify) | - Not stated - Details |
| Type of humanitarian emergency | - Not stated - Details |
| Funding details | - Not stated - Details |
| Was ethical approval gained? | - Not stated - Details |
| Are there any ethical concerns about the study? | - Not stated - Details |

| **Section C: Population** | |
| --- | --- |
| Age group (sample focus of the study if specified – for trials, specify who data is collected on. For process evaluations, specify the same, e.g. adults or children providing views as recipients. If the sample is collecting data from children – as peer deliverers of MHPSS still apply children and young people only) | - Children and young people only (children and young people aged 0–25 years or as specified in the paper) - Adults only (as specified in the paper) - Older people only (as specified in the paper) - No specific age group focus (if there is no age group focus or stated in the paper) |
| Other marginalized groups (as reported in the paper) | - Not applicable - Details |
| Gender | - Female only - Male only - No specific focus on gender |
| **Section D: Sample details** | |
| Study design | - Quantitative (please specify) - Qualitative (please specify) - Mixed-methods (please specify) |
| Sample focus | - Programme implementers/providers - Programme recipients |
| Sampling and recruitment methods (including recruitment)  *How were the subjects selected for the study?* | - Not stated - Details |
| Sample size | - Not stated - Details |
| Socio-demographic characteristics of participants | - Not stated - Details |
| **Section E: Data collection and analysis** | |
| Methods of data collection (please specify based on description in the paper) | - Not stated - Unclear - Survey - In-depth interviews - Semi-structured interviews - Participant observation - Focus groups - Diary study - Document analysis - Others (please specify) |
| Methods of data analysis (please specify based on description in the paper) | - Not stated - Unclear - Statistical analysis - Grounded theory - Framework analysis - Thematic analysis - Interpretative phenomenological analysis (IPA) - Others (please specify) |
| **Section F: Findings on process** | |
| Data/findings on contextual/ facilitators/barriers to intervention processes (extract findings including page numbers and if participation quotes, author description or author conclusions) | - Add themes/sub-themes - Feasibility - Fidelity - Accessibility - Acceptability - Satisfaction - Intensity/dose - Cultural sensitivity |
